# Aggregation of Modified Glucuronoxylan in Water and DMSO

**DOI:** 10.1002/bip.70091

**Published:** 2026-03-16

**Authors:** Chonnipa Palasingh, Ratchawit Janewithayapun, Leide P. Cavalcanti, Felix Abik, Kirsi S. Mikkonen, Fabrice Cousin, Anna Ström, Tiina Nypelö

**Affiliations:** ^1^ Department of Bioproducts and Biosystems Aalto University Espoo Finland; ^2^ Department of Chemistry and Chemical Engineering Chalmers University of Technology Gothenburg Sweden; ^3^ FibRe Center for Lignocellulose‐Based Thermoplastics Chalmers University of Technology Gothenburg Sweden; ^4^ ISIS Neutron and Muon Source Didcot UK; ^5^ Department of Food and Nutrition University of Helsinki Helsinki Finland; ^6^ Helsinki Institute of Sustainability Science University of Helsinki Helsinki Finland; ^7^ Laboratoire Léon Brillouin Université Paris‐Saclay, CEA Saclay 91191 Gif Sur Yvette France; ^8^ Wallenberg Wood Science Center Chalmers University of Technology Gothenburg Sweden

**Keywords:** beechwood xylan, hemicelluloses, SANS, uronic acid

## Abstract

Glucuronoxylans are known to be only partly soluble in aqueous media. Chemical modification often aims to improve solubility, yet observations of aggregation even of the modified xylans are not uncommon. We investigated the aggregation of glucuronoxylans of two different molar masses (XS and XM with *M*_w_ = 14 and 24 kg/mol, respectively), as well as their derivatives that were modified using periodate oxidation and borohydride reduction. Investigations were carried out in water and dimethyl sulfoxide (DMSO) by means of small angle neutron scattering (SANS). All dispersions of XS and its derivatives were turbid in water and translucent in DMSO. All samples based on XM were translucent in water and transparent in DMSO. In all cases, dispersions showed aggregates at the nanoscale with SANS, even for visually translucent and transparent dispersions with individual chains in a good solvent environment, indicated by the obtained Flory exponent of 0.588. Xylans dispersed in DMSO were less aggregated than xylan dispersed in water. The effect of solvent choice on the dispersibility of the modified xylans depended on the starting material composition. We propose that aggregation on the nanoscale is an intrinsic property of these polysaccharides and must be accounted for in processing, analysis, modification and applications.

## Graphical abstract

Neutron scattering study of dispersibility of glucuronoxylan and modified glucuronoxylan, in water and dimethyl sulfoxide (DMSO).

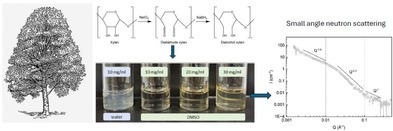

## 1 Introduction

The tendency toward polysaccharide aggregation owes primarily to the number of inter‐ and intra‐molecular bonding between the hydroxyl groups in the polymer chain and on the type and degree of substituents [1]. The type and degree of substituents of xylans, the main hemicelluloses found in hardwoods (10%–35%) and softwoods (10%–15%) [2] depend on the source and type of wood. 4‐*O*‐methylglucuronoxylans, that are xylans with 4‐*O*‐methyl‐alpha‐D‐glucopyranosyl uronic acids (MeGlcA) substitutions, are abundant in hardwoods [1, 3, 4, 5]; furthermore, they may contain acetyl groups at C2 and/or C3 of xylose backbone units [2]. Arabino‐4‐*O*‐methyl‐glucuronoxylans are commonly found in softwoods and are characterized by the presence of arabinose and lack of acetyl groups compared to the hardwood xylans [1, 2].

Xylans with low degree of arabinose form aggregates when dispersed in water [6, 7]. Acetylation of xylans, both at low and high degrees of substitution, also reduces the solubility of xylans in water [8, 9, 10], which positively influences the adsorption of xylans to cellulose [9, 11]. Entropic contribution stemming from the molar mass has been considered to also play a role in xylan solubility [12, 13], but the chemical composition is deemed more decisive for solubility than the molar mass [6, 8, 14]. The aggregation behavior of arabinoglucuronoxylans has been studied using light scattering and microscopy, showing poor solubility in water despite xylans carrying negative charges and dispersions being optically transparent [13, 15].

We have suggested that modification of the xylan backbone via periodate oxidation, followed by reduction can alter the xylan‐solvent interactions, and consequently, increase their solubility [12, 16]. Oxidation and reduction of polysaccharides are considered to introduce hinges in the backbone, thus increasing polymer flexibility. For example, oxidation of alginate, chitosan, and cellulose [17, 18] increases the chain compaction, as concluded based on reducing persistence lengths (*L*_p_) of the polysaccharides determined via size exclusion chromatography (SEC) equipped with multi‐angle light scattering (MALS) detector.

Here, we investigated solution properties of wood glucuronoxylans, and their oxidized and reduced derivatives in water and DMSO using small angle neutron scattering (SANS). SANS is a useful tool for investigating polymer conformation, as it probes several pertinent magnitudes of length‐scales of the system, enabling determination of the chain conformation and solvent interaction parameters. Water is a popular solvent choice for materials engineering, while water and DMSO are used as media for chemical reactions [19, 20, 21, 22] and for analytical determination of xylan molar mass (e.g., via SEC) and chemical composition via NMR.

## 2 Experimental

### 2.1 Materials

Beechwood xylans were purchased from Megazyme Ltd. (Ireland) and Sigma Aldrich (Sweden). The xylans are labeled in order of molar mass, as XM (medium) for the Megazyme xylan and XS (small) for the Sigma Aldrich xylan. Sodium periodate, sodium borohydride, ethylene glycol, dimethyl sulfoxide (DMSO), deuterium oxide (D_2_O), and DMSO‐*d*_6_ were acquired from Sigma Aldrich (Sweden). Pullulan standards were purchased from Postnova Analytics (Germany). The xylans and other chemicals were used without further purification.

### 2.2 Moisture and Ash Content

The moisture and ash content of xylans were analyzed by thermogravimetric analysis. Samples were heated from 20°C to 500°C under air flow, at a heating rate of 10 K/min. The moisture content was taken as the mass loss at 150°C, and the ash content was taken as the residual mass at 500°C.

### 2.3 Preparation of Dialcohol Xylan

The oxidation was done following the procedure adapted from Amer et al. [23] Xylan (4 g) was dispersed in Milli‐Q water (115 mL) overnight, then isopropanol (10 mL) was added to the xylan dispersion to act as a radical scavenger [24]. For the XS xylan, the process has been reported as well in Palasingh et al. [25]. The amount of xylan present in anhydroxylose units (AXU), where 1 AXU = 132.1 g/mol, was calculated for each xylan by subtracting its moisture and ash content, and the amount of NaIO_4_ to be added was then calculated, shown in Table 1. NaIO_4_ was dissolved in Milli‐Q water (75 mL) and added to the xylan dispersion. The reaction mixture was stirred at room temperature, protected from light, and terminated after 24 h by the addition of ethylene glycol (1 mL). The dialdehyde xylan product was purified by dialyzing against deionized water for 2 days. The DO was calculated by following the periodate consumption through its absorbance value at 290 nm using a UV–Vis spectrometer (Cary 60 UV–Vis Spectrophotometer, Agilent, United States) as described by Maekawa et al. [26].

**TABLE 1:** Amount of NaIO_4_ added for each oxidation experiment in comparison to the amount of xylan AXU.

Xylan used	Moisture content (wt%)	Ash content (wt%)	AXU added (mmol)	NaIO_4_ added
XS	6.6	11.9	24.8	3.3 g (0.62 equiv.)
XS	6.6	11.9	24.7	5.2 g (0.99 equiv.)
XM	3.7	6.6	27.2	3.5 g (0.60 equiv.)

*Note:* In all cases, 4 g of xylan was added, then the amount of NaIO_4_ needed was adjusted based on the dry content of each xylan material.

**FIGURE 1:**
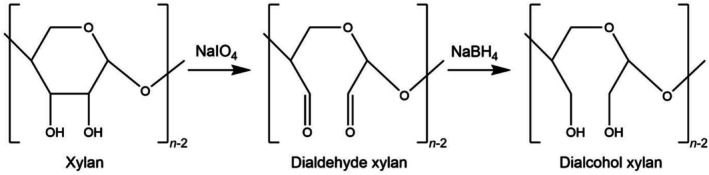
Schematic showing the periodate oxidation of xylan into dialdehyde xylan, followed by borohydride reduction into dialcohol xylan.

A borohydride reduction was employed to convert dialdehyde xylans to dialcohol xylans. The reduction was done at room temperature by addition sodium borohydride (2 g) for 2 days. Then, the products were dialyzed against deionized water and freeze‐dried for further analysis and storage. The reported yields were 49% for DalXS‐63, 30% for DalXS‐77 [25]. While the yield was not measured for DalXM‐60, previous oxidation–reduction of the same xylan gave yields of 40%–45% [16]. A schematic presentation of the modification of xylan is illustrated in Figure 1.

### 2.4 Carbohydrate Composition Determination

The carbohydrate composition of the xylans was determined by acid hydrolysis followed by anion exchange chromatography. The hydrolysis was done using sulfuric acid according to Theander and Westerlund [27]. In brief, 0.45 mL of 72% sulfuric acid was added to 30 mg xylan. The samples were placed under vacuum for 15 min, heated at 30°C for 1 h, then diluted with 12.6 g deionized water and autoclaved at 125°C for 1 h. The hydrolysates were filtered using glass microfiber filter before dilution to 25 mL total volume with deionized water. Solutions which contained 1 mL hydrolysate and 2 mL of 200 mg/L fucose standard were further diluted with deionized water with a 1:50 v/v ratio and were filtered through a 0.2 μm PVDF filter prior to the analysis. High performance anion exchange chromatography with pulsed amperometric detection (Dionex ICS‐3000, Dionex Corporation, United States) equipped with a CarboPac PA1 analysis column (Dionex Corporation, United States) was operated using NaOH and NaOAc as eluents.

### 2.5 Uronic Acid Content Determination

The amount of uronic acid in the xylans was determined by colorimetry using 3‐phenylphenol based on a method by Blumenkrantz and Asboe‐Hansen [28]. The detailed preparation of the reagents is described by Filisetti‐Cozzi and Carpita [29]. In brief, 10 mg xylan was hydrolyzed with cold 96% sulfuric acid (0.5 mL) and stirred using a vortex for 5 min before dilution with cold water (0.25 mL), followed by further vortex stirring. These steps were repeated one more time, resulting in a total volume of 1.5 mL. The system was then diluted with water to a total volume of 10 mL and further diluted with 9.6% sulfuric acid to a total volume of 30 mL. Eppendorf tubes that contained the diluted hydrolysate (160 μL), sulfamic acid‐potassium sulfamate (20 μL), and sodium tetraborate in sulfuric acid (12.5 mM, 800 μL) were heated to 95°C for 20 min. The coloring agent solution, 3‐phenylphenol (0.15% w/v, 40 μL), was added to the cooled solutions, and the samples' absorbance at 520 nm was measured with a UV–Vis spectrometer (Cary 60 UV‐Vis Spectrophotometer, Agilent, United States).

### 2.6 Molar Mass Determination

SEC was used to determine the molar mass of xylans and dialcohol xylans by dissolving in 0.01 M LiBr/DMSO at a concentration of 2 mg/mL. Xylans and dialcohol xylans were pre‐swollen with 30 μL Milli‐Q water overnight to enhance dissolution in LiBr/DMSO. The solutions were filtered using a 0.45 μm wwPTFE filter before they were injected into the SEC that was equipped with a Jordi xStream GPC column (JordiLabs, United States), and the systems were analyzed using refractive index and right‐angle light scattering (RALS, 670 nm, 90°) detectors. The temperature was 60°C at the column and 40°C at the detector. The flow rate was 0.8 mL/min.

### 2.7 Small‐Angle Neutron Scattering (SANS) Measurements and Data Analysis

Xylan and dialcohol xylan solutions were dispersed in deuterated solvents; D_2_O and DMSO‐*d*_6_ at concentrations between 10 to 30 mg/mL. In the case of DMSO‐*d*_6_, xylans and modified xylans were pre‐swollen in D_2_O (160 μL per 1 mL total volume) overnight prior to dispersion in DMSO‐*d*_6_. SANS data was collected at beamline SANS2D at ISIS Neutron and Muon Source, UK (RB2220239). The experiment was carried out using 12 m sample‐to‐detector distance and time‐of‐flight mode with wavelengths from 1.75 to 12.5 Å. Dispersions were placed in 2 mm path length quartz cuvettes, and loaded onto temperature‐controlled sample racks at 25°C.

Data averaging, transmission correction, and solvent subtraction were performed using the ISIS SANS Reduction GUI on the Mantid workbench 6.5. A polystyrene standard was used for absolute intensity calibration. Subtraction of incoherent scattering background and analysis of power law exponent of scattering curve were performed in MATLAB software. Fitting of data was performed in SasView 6.0.1 (https://www.sasview.org/) using the DREAM fitting algorithm.

## 3 Results and Discussion

### 3.1 Xylan Characterization

The weight‐average molar mass (*M*_W_) for the xylans is 14 and 24 kg/mol for the XS and XM, respectively (Table 2). The xylans were composed of xylose (≥ 96 wt%; Table 2). Other monosaccharides such as glucose, galactose, and arabinose were found in XM, while none were detected in XS. The uronic acid content was 7 wt% in XS and 11 wt% in XM.

**TABLE 2:** Carbohydrate composition, uronic acid content, weight‐average molar mass (*M*_W_), and molar mass dispersity (*Đ*) of xylans. Xyl, glu, gal, ara, man, rha are abbreviations for xylose, glucose, galactose, arabinose, mannose, and rhamnose, respectively.

Abbreviation	Relative carbohydrate composition (wt%)						Uronic acid (wt%)	*M*_W_ (kg/mol)	*Đ*
	Xyl	Glu	Gal	Ara	Man	Rha			
XS	93	0	0	0	0	0	7	14	2.5
XM	85	< 2	< 2	< 1	0	0	11	24	1.3

The DO of each modified xylan was determined by following the consumption of NaIO_4_ during the oxidation step (Table 3). The progress of the DO over the reaction period is also shown in Figure S1. For DalXM‐60, the UV–Vis scattering background shifts significantly and the absorbance after 24 h is lower than that of the reference XM in water. Therefore, it is assumed that all the periodate was consumed and the DO was taken as the maximum theoretical amount instead. The background subtraction and measurement by UV–Vis is subject to error from shifts in xylan solubility during the reaction and is further discussed by Palasingh et al. [16] where an NMR method for determining DO was compared. Overall, the UV–Vis method has been found to overestimate the DO of xylan by approximately 5%–10% units compared to NMR [16], in oxidized celluloses, this difference can be as large as 20% units [30].

**TABLE 3:** Degree of oxidation (DO), *M*_W_, and *Đ* of dialcohol xylans.

Abbreviation	Deriving from	DO (%)	*M*_W_ (kg/mol)	*Đ*
DalXS‐63	XS	63	12	2.8
DalXS‐77	XS	77	11	2.5
DalXM‐60	XM	60^a^	18	2.0

^a^ Since the UV–Vis background shifted, leading to negative absorbances after correction, the maximum theoretical DO was taken.

With higher achieved DO, a correlation was observed with increased degradation that was reflected in the molar mass (Table 3). The molar mass of XS was reduced by 14% and 22% in DalXS‐63 and DalXS‐77, respectively. The molar mass reduction of polysaccharides upon oxidation and reduction has been reported not only for 4‐*O*‐methylglucuronoxylans [31], but for other polysaccharides such as cellulose [32], chitosan [33], and alginate [34].

### 3.2 Visual Aspects of Xylan Dispersions

**FIGURE 2:**
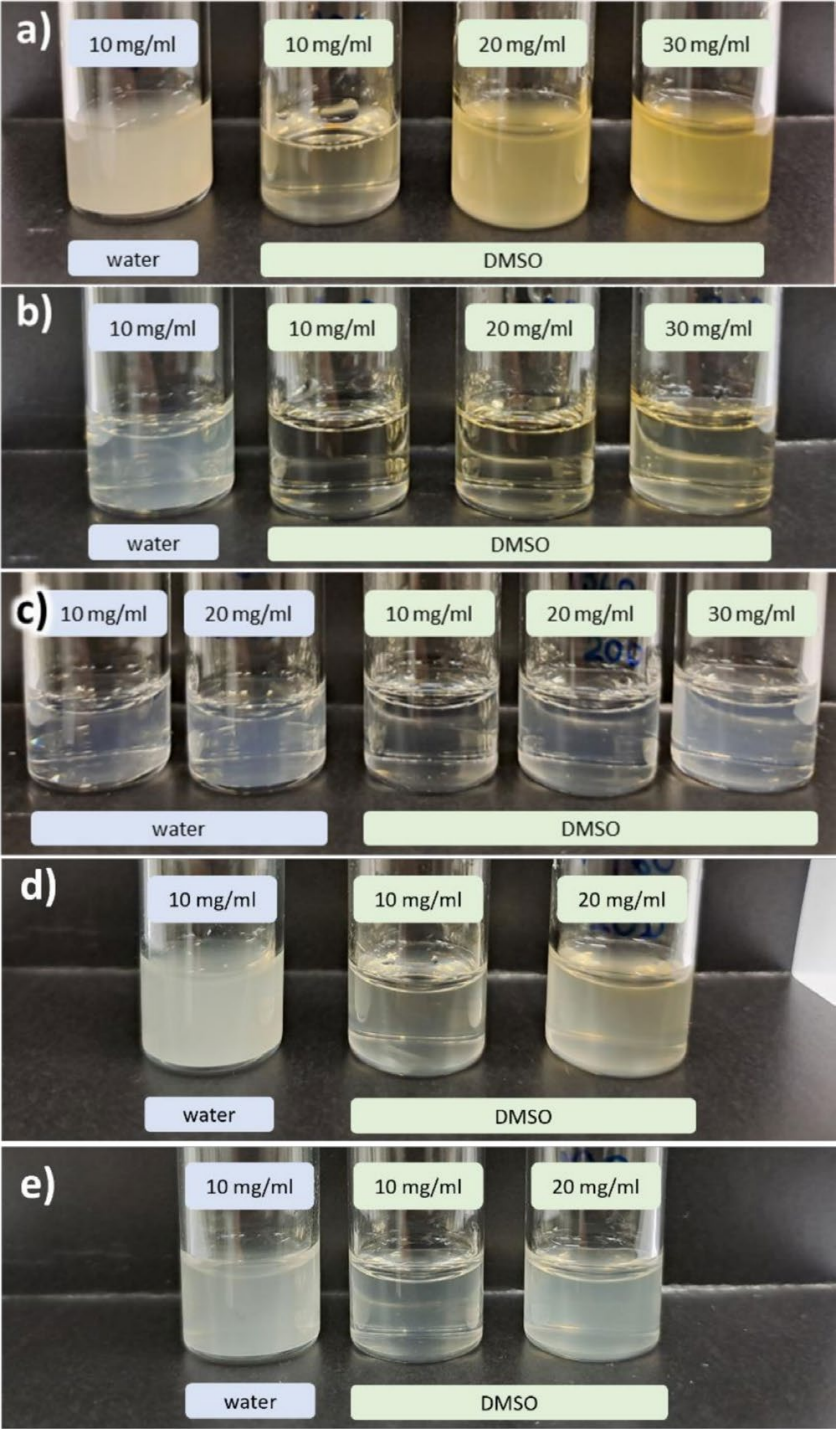
Photographs of XS (a), XM (b), DalXM‐60 (c), DalXS‐63 (d), and DalXS‐77 (e) dispersed in water and DMSO at concentrations of 10, 20, and 30 mg/mL. Examples of transparent dispersions are: XM in DMSO at 10–20 mg/mL. Translucent dispersions: DalXM‐60 in water at 10–20 mg/mL. Turbid dispersions: XS in water at 10 mg/mL, DalXS‐63 in water at 10 mg/mL.

By visual inspection, neither of the xylans produced a transparent dispersion in water at 10 mg/mL (Figure 2a,b for XS and XM, respectively). The extent of non‐transparency varied between the dispersions, with XS appearing turbid and XM being partially translucent. Dispersions of xylan in DMSO appear visually better dispersed for both XS (Figure 2a) and XM (Figure 2b), especially for XM, which forms transparent dispersions up to 20 mg/mL. This indicates good solvent interaction between XM and DMSO in line with findings reported by Ebringerová [3], Saake et al. [14] and reports that xylan generally disperse better in DMSO compared to water [35]. Oxidation and reduction of xylans result in a more translucent water dispersion, with the effect more visible in DalXM‐60 (Figure 2c) compared to DalXS‐77 (Figure 2e), on the other hand, DalXS‐63 (Figure 2d) did not show a signifōicant difference in turbidity. When the modified xylans were dispersed in DMSO, DalXM‐60 was less transparent above 10 mg/mL in comparison to XM. For both DalXS‐63 and DalXS‐77, their dispersions in DMSO remained translucent throughout, and the largest difference in comparison to XS was a more colorless appearance for the modified samples.

The size of individually dispersed xylan chains is below the sizes that scatter light, as judged by the molar mass (Table 3), hence the non‐transparent dispersions indicate presence of micro‐sized aggregation of xylan chains. From comparisons of XS and XM, we observe that the molar mass is not indicative of their tendency to aggregate. The higher number of the substituents indicates a more branched structure of XM in comparison to XS (Table 2) and this may be the differentiating factor for their solubility as observed by the visual inspection. It should be noted, however, that the refractive index of DMSO is higher than that of water, which may affect visual appearances of any aggregates.

### 3.3 Conformation and Sizes of Structures of Xylans in Water Dispersions

**FIGURE 3:**
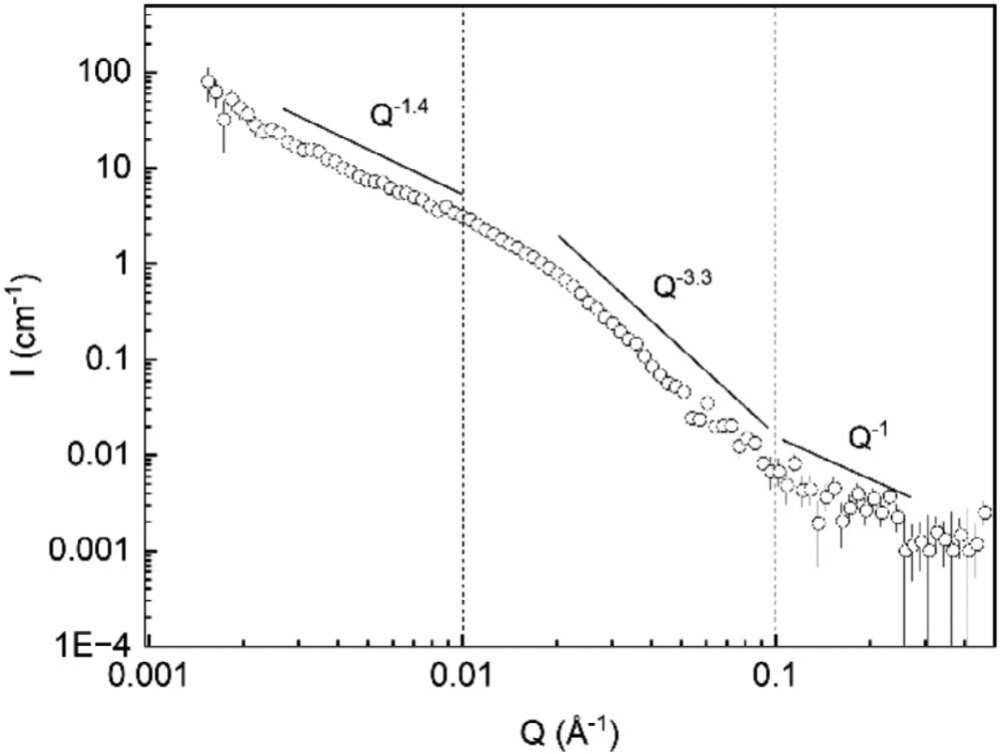
SANS data of the XM dispersion in D_2_O at a concentration of 10 mg/mL.

Only the XM water dispersion was translucent and hence of interest for SANS investigation. The SANS curve for 10 mg/mL XM in deuterated water (D_2_O) is shown in Figure 3. The SANS scattering curve was divided into high, intermediate, and low *Q* regions for analysis, corresponding to *Q* > 0.1 Å^−1^, 0.01 < *Q* < 0.1 Å^−1^ and *Q* < 0.01 Å^−1^, respectively. In the high *Q* region, a *Q* ^−1^ power law scaling of the scattering was observed, which is characteristic of a polysaccharide with stiff rod‐like segments [36]. The scattering intensity shows a transition from the *Q* ^−1^ scaling around Q* = 0.1 Å^−1^ from which an estimate of the apparent persistence length (*L*_p_) can be obtained as *L*_p_ = 6/πQ* [37]. The value obtained is only the apparent *L*_p_ as local aggregations can influence the observed *L*_p_. The apparent *L*_p_ for XM in water would then be approximately 2 nm, which is rather short compared to that reported for polysaccharides such as xyloglucan (8 nm) [36] and wheat arabinoxylan (4.5 nm) [38].

A scaling exponent of *Q* ^−3.3^ was observed at intermediate *Q*, which means that we are observing scattering from a surface fractal of a dense and compact 3D structure [38, 39]. In the intermediate *Q* region, the size of structures that are probed are those with a radius of gyration (*R*_g_) around 10 nm in direct space, corresponding to the Guinier regime (QR_g_) < 1 [36, 39]. The compact structure is then likely to be a cluster of a few tightly packed xylan chains.

At low *Q* values, a *Q* ^−1.4^ decay was observed and related to the internal fractal dimension of larger aggregates formed from smaller clusters. Hence, it is plausible that the compact clusters of XM aggregate at larger length scales to form open structures with low compactness and larger sizes. Larger here meaning that the sizes of the structures are outside the *Q*‐range of our experiment (lowest *Q* is 0.002 Å^−1^), which corresponds to the Guinier regime size limit (from QR_g_ < 1) of particles of sizes approximately 30–50 nm.

The visual appearance and SANS analyses confirm poor dispersibility of glucuronoxylan in water. The individual XM chains were in a poor solvent environment and existed as collapsed chains, forming dense and compact poorly solvated clusters of radius ~10 nm that aggregate at larger scale in open fractal aggregates reaching sufficiently large sizes to scatter light (> a few hundreds of nm). The trend is similar to those found for arabinoglucuronoxylans using light scattering and the Flory–Huggins theory [13].

### 3.4 Conformation and Sizes of Structures of Xylans in DMSO Dispersions

**FIGURE 4:**
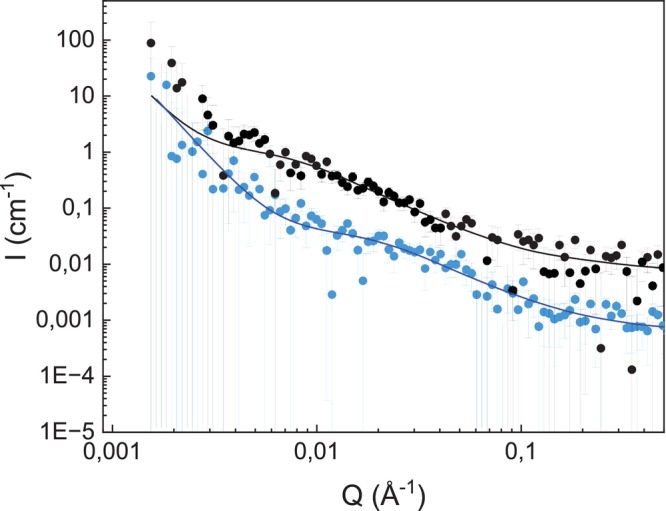
SANS data, with fits to the flexible cylinder model (lines), of XS (blue) and XM (black) xylan dispersions in DMSO‐*d*_6_ at a concentration of 10 mg/mL. The intensity has been shifted by a factor of 5 for XM for ease of viewing.

SANS measurements were performed for XS and XM at concentrations of 10, 20, and 30 mg/mL in DMSO‐*d*_6_. The scattering profiles from 10 mg/mL (Figure 4) and 20 mg/mL dispersions (Figure S2) were similar, while the scattering of the 30 mg/mL dispersions becomes notably different (Figure S3). At 10 mg/mL, the scattering curve demonstrated four distinct features: (i) the *Q* ^−1^ scaling from the rod‐like segments in the high *Q* region is observed for XS and XM, like when the XM were dispersed in water. The apparent *L*_p_ can be estimated in the same way, with the DMSO‐*d*_6_ transition *Q* ≈ 0.08 Å^−1^ (Figure 4) for XS and XM to give *L*_p_ ≈ 2.4 nm, slightly longer than the *L*_p_ of XM in water. A comparison of XS and XM in a Q^1^I plot is also given in Figure S4; (ii) in the intermediate *Q* region, the SANS curve exhibited a power law decay with exponents around 1.8; (iii) preceded by a pronounced shoulder and a plateau at around 0.008–0.02 Å^−1^; and (iv) in the low *Q* region, the curve showed a gradually increasing upturn toward low *Q*, ending in a scaling of Q^−4^.

For polymers in solution in the dilute regime, the scattering pattern at the intermediate *Q* region enables probing of the behavior of the chains and the Flory exponent (ν). With *I*(*Q*) decaying with *Q* ^−1/ν^. At intermediate *Q*, for both XS and XM, the decay is *Q* ^−1.8^, which gives *ν* = 1/1.8 = 0.56, indicating the chains are swollen and in the good solvent regime [39, 40]. At lower *Q*, a *Q* ^−4^ scaling is again observed for both XS and XM. We assign the *Q* ^−4^ upturn to the surface scattering (Porod scattering) of large, compact structures; hence, the xylan dispersions contained dense aggregates (> > 50 nm) that coexist with well‐dispersed chains, despite XS and XM being in a good solvent environment.

The scattering intensity of XS and XM at 10 mg/L in DMSO was fitted to a flexible cylinder model in good solvent conditions, with an added power law term accounting for aggregate contributions. Due to low measurement statistics at high *Q* and to minimize the number of fit parameters, we do not fit the chain cross‐section radius; instead, we fix the chain cross‐section radius to 5 Å, corresponding to the size of one xylose residue. The resulting fits are shown in Figure 4. Here, we report best fit values with the 68% confidence interval within brackets [lower limit, upper limit]. The *L*_p_ (where *L*_p_ = Kuhn length/2) from both fits is indeed short, at 0.9 nm [0.4 nm, 1.9 nm] for XS and 1.5 nm [0.8 nm, 2.6 nm] for XM, in agreement with estimates from the model independent approach. The *R*_g_ was calculated from the contour length [41] as 7 nm [5 nm, 11 nm] for XS, and 18 nm [15 nm, 25 nm] for XM. From the volume fraction obtained from modeling with the flexible cylinder model in absolute scale, taking into account the scattering length densities of xylan and solvent, we also obtain an estimate of the volume fraction of XS and XM observed as dispersed chains in the sample. These volume fractions were, respectively, 0.0006 [0.0003, 0.0009] for XS and 0.0009 [0.0006, 0.0014] for XM. Taking the specific partial volume of chains as 0.6 cm^3^/g [42], the volume fraction corresponds to concentrations of dispersed chains of 1.0 and 1.5 mg/mL, respectively, for XS and XM, out of the total of 10 mg/mL added to DMSO. Therefore, we reason that between 10% to 20% of xylan chains are dispersed at 10 mg/mL, with a higher population for XM than XS. The rest of the XS and XM exist as aggregates dispersed in DMSO.

When the concentration was increased to 20 mg/mL (Figure S2), the scattering curve features are similar, but in the intermediate *Q* region, we observe aggregation of the dispersed xylan chains on the level of a few xylan chains. This is indicated by a shift of the shoulder around *Q* = 0.01 Å^−1^ shifts to lower Q, as well as an increase in intensity of the region, relative to concentration. When increasing the concentration to 30 mg/mL (Figure S3), XS and XM showed an increased power law exponent in the intermediate *Q* region to *Q* ^−2.3^. The increased power law exponent at intermediate *Q* indicates a decrease in solvent quality and partial desolvation of the chains and therefore an increase in intermolecular interaction as a function of concentration. The tendency of xylan to aggregate could be an intrinsic property due to the molecular structure of xylans, as already suggested for arabinoxylan [38, 43].

XS and XM appeared to be in good solvent conditions in DMSO‐*d*_6_, in line with current practices that DMSO acts as a better solvent for xylans than water. However, despite being in a good solvent, there remain dense aggregates in all xylan suspensions, with sizes larger than those probed in the q‐range window of the SANS experiment (50 nm in direct space).

### 3.5 Dialcohol Xylans Dispersed in Water and in DMSO

**FIGURE 5:**
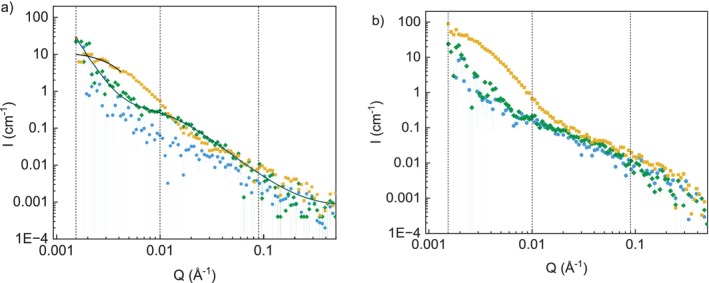
SANS data of XS (blue circle), for reference, and dialcohol XS with DO of 63% (DalXS‐63; green diamond) and 77% (DalXS‐77; yellow square) dispersed in DMSO‐*d*_6_, at a concentration of 10 mg/mL. Lines represent a polymer chain with excluded volume fit for DalXS‐63, and a Guinier fit for DalXS‐77 (a). SANS data of the same samples at a concentration of 20 mg/mL are shown in (b).

SANS measurements were performed for DalXS‐63 and DalXS‐77 at concentrations of 10 and 20 mg/mL in DMSO‐*d*_6_. The SANS curve of DalXS‐63 in DMSO‐*d*_6_, 10 mg/mL is shown in Figure 5a. In the high Q region, the scattered curve still followed the same *Q* ^−1^ scaling, like the non‐modified XS. Within error margins, there was no change in the *Q* values where the intensity deviated from a Q^−1^ scaling in the intermediate *Q* region; therefore, there was no significant change in *L*_p_ of the modified XS. The shoulder for DalXS‐63 is shifted to lower *Q* compared to XS, at around *Q* = 0.007 Å^−1^.

A fit to the polymer chain with excluded volume model [44] was made, giving an *R*_g_ of 12 nm and a power law exponent of *Q* ^−2.1^. The chains of DalXS‐63 are lower in *M*_w_ than XS, therefore a higher *R*_g_ as obtained for the XS (7 nm), indicates formation of aggregates of a few DalXS‐63 chains. The increase in the power law exponent reflects an increase in mass density, which can come from a change in solvent interaction and/or aggregation. When the concentration of DalXS‐63 was increased to 20 mg/mL (Figure 5b), the scattering almost superimposes with that of the XS sample at 20 mg/mL at intermediate and large *Q*. The transition between the *Q* ^−4^ decay at low *Q* associated to aggregates, and the *Q* ^−2.3^ decay scattering of the isolated solvated chains has been shifted toward large *Q*, showing that the contribution from aggregates to the scattering becomes more significant, that is, the aggregates are larger and/or more numerous at 20 mg/mL. The oxidation step has thus slightly increased aggregation of chains with increased concentration. The shift of the *Q* ^4^–Q^−2.3^ crossover does not show a clear Guinier plateau, thus determination of the *R*_g_ of the aggregate becomes uncertain and was not attempted.

In the case of DalXS‐77 in DMSO‐*d*_6_ (Figure 5a,b), the scattering profile was comparable to XS and DalXS‐63 in the high *Q* region. While at low *Q*, the scattering resembles that of the form factor of polydisperse compact spheres of small radius, with a Guinier plateau at the lowest *Q* followed by a *Q* ^−4^ decay with increasing *Q* values. The Guinier plateau was fitted with a Guinier model to obtain an *R*_g_ of 46 nm. Given the compact nature of this larger aggregate, the overall scattering was fitted by summing a spherical form factor with lognormal polydispersity of the radius to a flexible cylinder model (see Figure S5 and Table S1).

The fitting allows for an estimation of the order of magnitude of the volume fraction of the spheres and of the flexible cylinder chains. The scale factor of the scattering intensity from the spheres in solutions (*K*_spheres_) is related to its volume fraction (Φ_spheres_) by Φ_spheres_ (*ρ*_spheres_ − *ρ*_solvent_)^2^. The parameter *ρ*_spheres_ is an effective contrast of the spherical aggregates, wherein both xylan chains and solvent are present, and takes into account the inner volume fraction of chains (Φ_inner_) within the aggregates. This contrast is given by *ρ*_spheres_ = Φ_inner_ *ρ*_xylan_ + (1 − Φ_inner_) *ρ*_solvent_. *K*_spheres_ then writes Φ_spheres_ Φ_inner_^2^ (*ρ*_xylan_ − *ρ*_solvent_)^2^, allowing for the determination of the magnitude of the volume fraction of aggregates Φ_spheres_ from *ρ*_xylan_ and ρ_solvent_. Assuming that Φ_inner_ is larger than ~0.3, as the aggregates would be too swollen to give rise to a Porod scattering with *Q* ^−4^ decay otherwise, we calculate that Φ_spheres_ is on the order of magnitude of 10^−5^. This volume fraction can be compared to the volume fraction of dispersed flexible chains, which are on the order of 10^−3^.

Therefore, we conclude that, of the structures visible in SANS, DalXS‐77 exists as dispersed chains, with a small population aggregating to form larger structures with an *R*_g_ of 46 nm. Increasing concentration from 10 to 20 mg/mL resulted in an increase in the number of DalXS‐77 chains in the aggregates. Overall, the conclusion is that modification reduces the individual dispersibility of the xylan chains in both DalXS‐63 and DalXS‐77, resulting in a small population of the dispersed xylans interacting to form aggregates.

**FIGURE 6:**
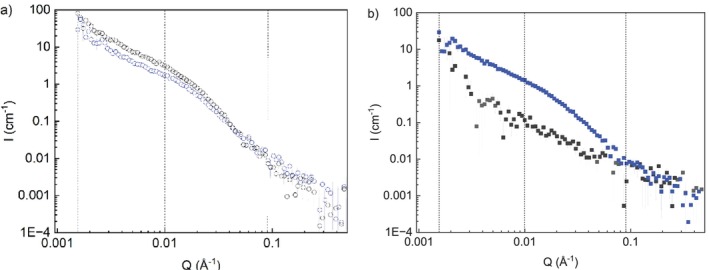
SANS data of XM (black), for reference, and DalXM‐60 (blue) at a concentration of 10 mg/mL dispersed in D_2_O (a) and DMSO‐*d*_6_ (b).

When dispersed in DMSO, DalXM‐60 was transparent at 10 mg/mL but became more turbid with increasing concentration, similar to XM in DMSO. The scattering curve for DalXM‐60 in D_2_O had similar features as that of XM in D_2_O (Figure 6a), showing that structures are comparable in both solvents. The only difference concerns the power law exponent of the intermediate *Q* region of DalXM‐60 at 10 mg/mL that was reduced compared to the D_2_O dispersion: The XM in D_2_O had an exponent of *Q* ^−3.3^, whereas the DalXM‐60 had an exponent of *Q* ^−2.7^, indicating that DalXM‐60 became less compact after modification and interacts better with water, in line with faster dispersibility and the appearance of the dispersion of DalXM‐60 being more translucent than unmodified XM in water at 10 mg/mL (Figure 2). However, a *Q* ^−1.4^ exponent, correlated to larger aggregate structures, was still observed in the low Q region. Since the general features of the low Q scattering curve were similar to XM, the system appeared to form similar aggregate structures after modification.

In the case of DMSO*‐d*_6_, the scattering pattern of DalXM‐60 changed significantly in comparison to the unmodified XM (Figure 6b). The power law decay increased to *Q* ^−2.7^, compared to *Q* ^−1.8^ for XM in the intermediate *Q* region. While the latter indicates scattering from a chain in a good solvent, an exponent of *Q* ^−2.7^ indicates the opposite, suggesting that the DalXM‐60 had a compact conformation approaching that of a collapsed chain when dispersed in DMSO‐*d*_6_. At lower *Q* values, the same *Q* ^−1.4^ decay was observed, showing the aggregate structure was similar to DalXM‐60 and XM in D_2_O.

The influence of modification and DO of xylans to dispersibility depended on the properties of the starting xylan. XS derivatives showed no difference in terms of the visual appearances of the dispersions in water nor DMSO, and no improvements were observed by SANS. The modified XM, which was a more substituted xylan from the beginning, showed changes (solvent interactions) after the modification. The interaction of DalXM‐60 and water was improved, but interaction with DMSO was worsened. A summary of the behaviors of xylans and their derivatives is shown in Table 4.

**TABLE 4:** Summary of the characteristics of xylans; XS and XM, and dialcohol xylans; DalXS‐63, DalXS‐77, and DalXM‐60 in dispersion.

	Solvent	Dispersibility
XS	DMSO	Translucent dispersion. SANS shows chains in a good solvent with *R*_g_ = 7 nm mixed with larger aggregates.
	Water	Turbid, poorly dispersed.
DalXS‐63	DMSO	Translucent dispersion. SANS shows chains in a good solvent. Some small aggregates with *R*_g_ = 12 nm.
	Water	Turbid, poorly dispersed.
DalXS‐77	DMSO	Translucent dispersion. SANS shows chains in a good solvent. Minor population of larger aggregates with *R*_g_ = 46 nm.
	Water	Turbid, poorly dispersed.
XM	DMSO	Transparent dispersion. SANS shows chains in a good solvent with *R*_g_ = 17 nm mixed with larger aggregates.
	Water	Translucent dispersion. SANS shows dense compact clusters of xylan chains.
DalXM‐60	DMSO	Transparent dispersion. SANS shows poorer solvent interaction than XM, resulting in small clusters of DalXM.
	Water	Translucent, clearer than XM. SANS shows improved solvent interaction compared to XM.

## 4 Conclusions

Probing xylans in water and DMSO using SANS showed that the conformation of xylan chains is affected by the solvent choice. Xylans collapse in water (poor solvent conditions) and are more extended in DMSO (flexible chain in good solvent conditions). Even if the xylan behaves as flexible chains in good solvent condition when dispersed in DMSO, significant quantities of xylan aggregates are always observed in SANS, even for the visually transparent dispersions at low xylan concentrations. The extent of aggregation is increasing with higher xylan concentrations.

The effect of oxidation and reduction on solvation of XS and XM in water and DMSO was complex. Regardless of modification, XS formed turbid dispersions in water while the modified XM (DalXM‐60) appeared better dispersed in water. In DMSO, the modification did not change the solvent interactions for DalXS‐63 and DalXS‐77 and did not improve dispersibility. For the XM, which was seemingly well dispersed in DMSO, the oxidation and reduction modification resulted in worsened solvent interactions, while the solvent interaction in water was improved. The findings contribute to a better understanding of xylan dispersibility in water and DMSO, two solvents that are commonly used for analytics of polysaccharides. Dispersibility in water is of importance in the adsorption and precipitation of xylans in the pulp and paper industry. For instance, moderate aggregation can increase the adsorption of xylans on cellulose and help in controlling process yields.

## Funding

This work was supported by VINNOVA (2019‐03607), Vetenskapsrådet (2017‐05138), Nordisk Ministerråd (SNS Hemisurf 127), Svenska Forskningsrådet Formas (2020‐01235), Science and Technology Facilities Council.

## Conflicts of Interest

The authors declare no conflicts of interest.

## Supporting information


**Figure S1:** Plot of the degree of oxidation (DO) in % as a function of reaction time for DalXM‐60 (blue), DalXS‐63 (orange), and DalXS‐75 (green). Dashed lines show the theoretical maximum DO based on the amount of NaIO_4_ added to the reaction.
**Figure S2:** SANS scattering of XS (blue) and XM (black) dispersed in DMSO‐*d*_6_ at concentration of 20 mg/mL.
**Figure S3:** SANS scattering of XS (blue circle) and XM (black square) dispersed in DMSO‐*d*_6_ at concentration of 30 mg/mL.
**Figure S4:** SANS Q^1^I plot for XS (blue) and XM (black) xylan dispersions in DMSO‐*d*_6_ at concentration of 10 mg/mL. The intensity has been shifted by a factor of 5 for XM for ease of viewing.
**Figure S5:** DalXS‐75 in DMSO‐*d*_6_ at concentration of 10, lines show fitting of a spherical model with lognormal polydispersity of the radius added to a flexible cylinder model.
**Table S1:** Fitted parameters for DalXS‐75 in DMSO‐*d*_6_ at concentration of 10 mg/mL for a spherical model with lognormal polydispersity added to a flexible cylinder model. Reduced chi‐squared = 1.08.

## Data Availability

The data that support the findings of this study are available from the corresponding author upon reasonable request.
