# Supplementary material for: Aggregation of Modified Glucuronoxylan in Water and DMSO
Source: Biopolymers. 2026 Mar 16;117(3):e70091. doi: 10.1002/bip.70091 (PMC12993250; doi:10.1002/bip.70091)
Supplement: Supplementary file 1 — Figure S1: Plot of the degree of oxidation (DO) in % as a function of reaction time for DalXM‐60 (blue), DalXS‐63 (orange), and DalXS‐75 (green). Dashed lines show the theoretical maximum DO based on the amount of NaIO4 added to the reaction. Figure S2: SANS scattering of XS (blue) and XM (black) dispersed in DMSO‐d 6 at concentration of 20 mg/mL. Figure S3: SANS scattering of XS (blue circle) and XM (black square) dispersed in DMSO‐d 6 at concentration of 30 mg/mL. Figure S4: SANS Q1I plot for XS (blue) and XM (black) xylan dispersions in DMSO‐d 6 at concentration of 10 mg/mL. The intensity has been shifted by a factor of 5 for XM for ease of viewing. Figure S5: DalXS‐75 in DMSO‐d 6 at concentration of 10, lines show fitting of a spherical model with lognormal polydispersity of the radius added to a flexible cylinder model. Table S1: Fitted parameters for DalXS‐75 in DMSO‐d 6 at concentration of 10 mg/mL for a spherical model with lognormal polydispersity added to a flexible cylinder model. Reduced chi‐squared = 1.08. [file BIP-117-e70091-s001.docx]

**Supporting information for**

**Aggregation of modified glucuronoxylan in water and DMSO**

**Chonnipa Palasingh^1,2, ^^, Ratchawit Janewithayapun^2, ^^, Leide P. Cavalcanti^3^, Felix Abik^4^, Kirsi S. Mikkonen,^4,5^ Fabrice Cousin,^6^ Anna Ström^2*^, Tiina Nypelö^1*,2,7^**

^1^ Department of Bioproducts and Biosystems, Aalto University, 00076 Aalto, Finland

^2^ Department of Chemistry and Chemical Engineering, Chalmers University of Technology, 41296 Gothenburg, Sweden

^3^ISIS Neutron and Muon Source, STFC, OX110QX, Didcot, United Kingdom

^4^Department of Food and Nutrition, 00014 University of Helsinki, Finland

^5^Helsinki Institute of Sustainability Science, 00014 University of Helsinki, Finland

^6^Laboratoire Léon Brillouin, Université Paris-Saclay, UMR 12, CEA-CNRS, 91191 Gif Sur Yvette, France

^7^Wallenberg Wood Science Center, Chalmers University of Technology, Gothenburg, Sweden

*corresponding authors: [anna.strom@chalmers.se](mailto:anna.strom@chalmers.se), [tiina.nypelo@aalto.fi](mailto:tiina.nypelo@aalto.fi)

^The authors contributed equally


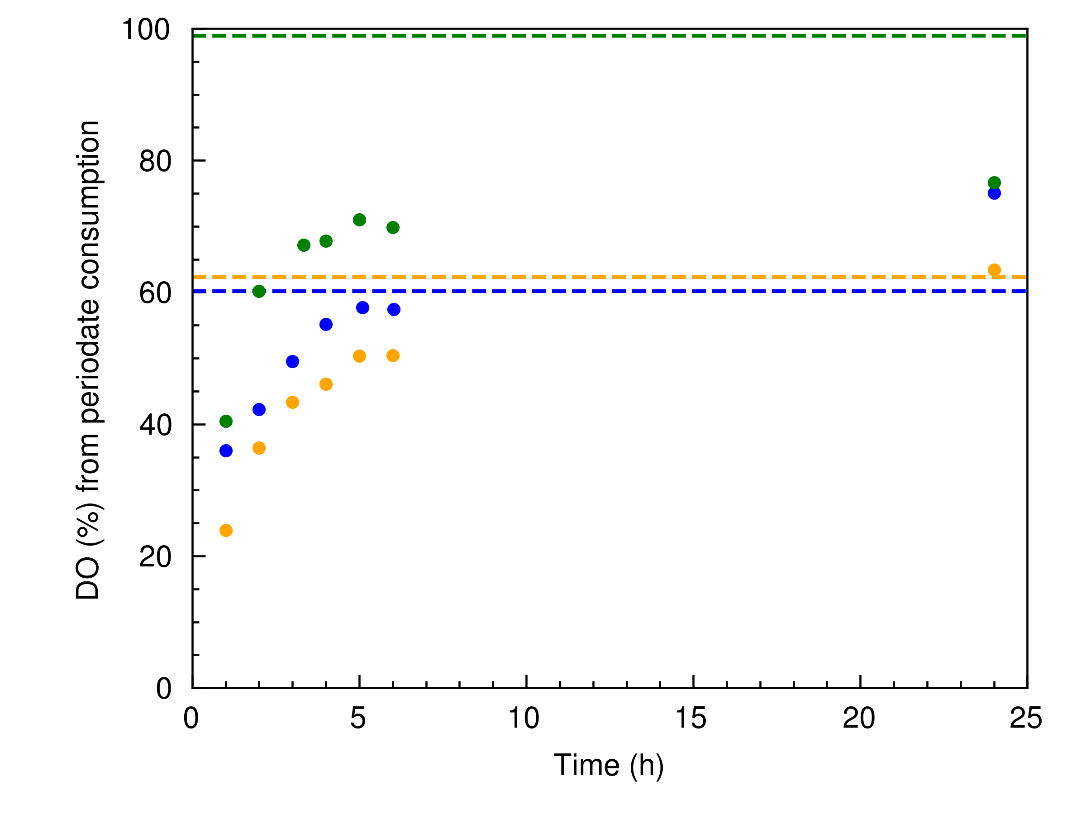


Figure S1. Plot of the degree of oxidation (DO) in % as a function of reaction time for DalXM-60 (blue), DalXS-63 (orange), and DalXS-75 (green). Dashed lines show the theoretical maximum DO based on the amount of NaIO_4_ added to the reaction.


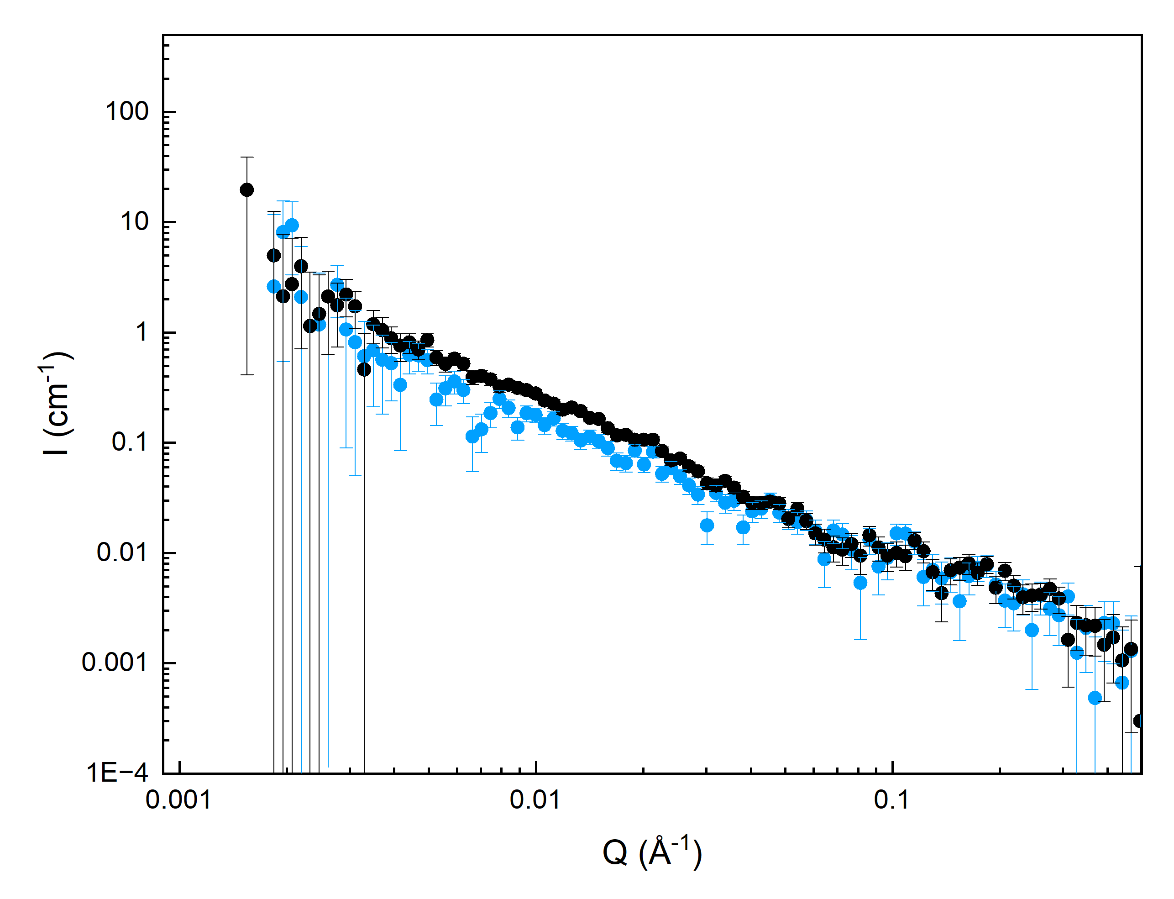


Figure S2. SANS scattering of XS (blue) and XM (black) dispersed in DMSO-*d_6_* at concentration of 20 mg/mL.

Figure S3. SANS scattering of XS (blue circle) and XM (black square) dispersed in DMSO-*d_6_* at concentration of 30 mg/mL.

Figure S4. SANS Q^1^I plot for XS (blue) and XM (black) xylan dispersions in DMSO-*d_6_* at concentration of 10 mg/mL. The intensity has been shifted by a factor of 5 for XM for ease of viewing

Figure S5. DalXS-75 in DMSO-*d6* at concentration of 10, lines show fitting of a spherical model with lognormal polydispersity of the radius added to a flexible cylinder model.

Table S1. Fitted parameters for DalXS-75 in DMSO-*d6* at concentration of 10 mg/mL for a spherical model with lognormal polydispersity added to a flexible cylinder model. Reduced chi-squared = 1.08.

|  | **Value** | **Error** | **Unit** |
| --- | --- | --- | --- |
| **Background** | 2×10^-3^ | - | cm^-1^ |
| **Flexible chain volume fraction** | 1×10^-3^ | 3.90×10^-5^ |  |
| **Chain length** | 6×10^2^ | - |  |
| **Kuhn length** | 2×10^1^ | - |  |
| **Cross-section radius** | 6 | - |  |
| **SLD chain** | 2.36 | - | 10^-6^/ Å^2^ |
| **SLD solvent** | 5.28 | - | 10^-6^/ Å^2^ |
| **Sphere volume fraction** | 4.97×10^-5^ | 1.02×10^-6^ |  |
| **SLD sphere** | 2.36 | - | 10^-6^/ Å^2^ |
| **SLD solvent** | 5.28 | - | 10^-6^/ Å^2^ |
| **Sphere radius** | 1.60×10^2^ | 7.8 | Å |
| **Radius polydispersity (lognormal)** | 4.40×10^-1^ | 2.41×10^-2^ |  |
